# Do Face-to-Face Training and Telephonic Reminder Improve Dry Powder Inhaler Technique in Patients with COPD?

**DOI:** 10.1155/2017/5091890

**Published:** 2017-03-13

**Authors:** Ramesh Sharma Poudel, Shakti Shrestha, Pawan Bhatta, Rano Mal Piryani

**Affiliations:** ^1^Hospital Pharmacy, Chitwan Medical College Teaching Hospital, Chitwan, Nepal; ^2^Department of Pharmacy, Shree Medical and Technical College, Chitwan, Nepal; ^3^Department of Pharmacy, Nobel College of Medical Science, Kathmandu, Nepal; ^4^Department of Internal Medicine, Chitwan Medical College Teaching Hospital, Chitwan, Nepal

## Abstract

*Introduction*. Current modes of instruction on inhaler technique are inadequate. We aimed to evaluate the value of face-to-face training and telephonic reminder (FFTTR) for improving Rotahaler technique in experienced patients with COPD.* Materials and Methods*. A single group pre-/postinterventional study was conducted at Chitwan Medical College Teaching Hospital, Nepal. We assessed the Rotahaler technique of thirty consecutive patients using Rotahaler device for more than one year. Patients with incorrect technique (*n* = 20) were instructed and trained by a pharmacist. Telephonic reminder was used to reinstruct patients on the correct technique on weekly basis for two weeks and technique was reassessed after 4 weeks of their first training. Descriptive statistics including Wilcoxon Signed Rank test were applied.* Results*. The mean age was 66.06 ± 10.6. Of 30 patients, 10 (33.3%) performed Rotahaler technique correctly at baseline and were excluded from FFTTR intervention. FFTTR corrected the technique in 18 (90%) patients and the median (IQR) score increased from 6 (5-6) to 8 (8-8) (*p* < 0.001). The most incorrect steps were “breathe out gently but not towards the inhaler mouthpiece” (16, 80%) and “hold breath for about 10 seconds” (18, 90%) at baseline which improved after intervention.* Conclusion*. FFTTR approach markedly improved Rotahaler technique in patients with COPD.

## 1. Introduction

COPD accounts for 43% of the total noncommunicable diseases (NCDs) in Nepal [1]. Such patients are principally treated with inhaled medications using inhaler devices, preferentially pressurized metered dose inhalers (pMDIs) and dry powder inhalers (DPIs) [2]. Correct inhalers technique is indispensable for better treatment outcomes and for control of disease [3]. Studies have shown that 58.8 to 100% of Nepalese patients with COPD had incorrect inhalers technique [4–6] and up to 61.1% of patients had never received instruction on correct use of inhalers [4, 6]. Such inadequate technique can result in reduced therapeutic effects, poor control of symptoms, and therefore inadequate disease management [7]. Furthermore, insufficient inhaler technique has been associated with an increased risk of hospital admission, emergency department visits, and courses of oral steroids and antimicrobials [3, 8]. Nepalese patients living with COPD have experienced physical and psychosocial health problems in their daily life [9], possibly due to poor disease control contributed by improper inhalers technique. In Nepal, hospital pharmacy services are at primitive stage and, therefore, pharmaceutical care is inadequate [10, 11]. Moreover, healthcare professionals (HCPs) also lack enough knowledge on correct use of inhalers technique [12–16]. The reported methods of instruction/intervention used for the improvement of different inhalers technique in Nepalese patients with asthma and COPD include physical demonstration; combination of verbal instruction and physical demonstration; combination of verbal counseling, physical demonstration, and video demonstration; and combination of verbal instruction, physical demonstration, and face-to-face training [4, 6, 17, 18]. Evidences suggest that these modes of instruction have absolutely corrected the inhalers technique in 3.3 to 70% of the patients depending on the types of inhalers used [4, 6, 17–19]. This indicates that the currently used methods of instruction are inadequate for the optimization of inhalers technique in these groups of patients and indeed highlights the necessity of additional intervention for improving inhalers technique. Our pilot study aimed to evaluate the value of FFTTR for improving Rotahaler technique in experienced patients with COPD.

## 2. Materials and Methods

A single group pre-/postinterventional study was conducted at the Medication Counseling Centre of Chitwan Medical College Teaching Hospital, Bharatpur 10, Chitwan, Nepal, from October to November 2015. Ethical approval of this study was obtained from the Institutional Review Committee of the Chitwan Medical College Teaching Hospital. Clinically diagnosed patients with COPD attending the outpatient pharmacy and using Rotahaler device for more than one year (experienced patients) were considered eligible for this study. The patients were consecutively enrolled. Patients using Instahaler, Lupihaler, and Revolizer; suffering from dementia or other neurological deficits; using Rotahaler for <1 year; unwilling to participate; and lost to the follow-up were excluded. Patients were well informed about the study, and verbal informed consent was obtained from all patients before participation in the study as majority of patients were illiterate.

We recruited two registered pharmacists for this study who were educated and trained in DPIs technique by the Health Professionals Education and Research Centre of Chitwan Medical College Teaching Hospital. In addition to this, pharmacists were instructed to use drug information leaflets enclosed inside the Rotacaps and Rotahaler device. The first pharmacist conducted the structured interview on a total of thirty patients to obtain baseline information. These included age, sex, educational status, duration of Rotahaler use, previous instruction on Rotahaler technique, sources of instruction, methods of instruction, repeated instruction, number of hospital visits for the last 12 months, antimicrobial courses for the last 12 months, emergency department visits for the last 12 months, and hospitalization for the last 12 months. Then patients were requested to demonstrate their technique using placebo, while the pharmacist made the initial assessments of technique as per the 8-item Rotahaler specific checklist developed in our previous study [18]. Steps  1, 3, and 6 were considered critical [20]. Each correct step was scored “1” and an incorrect or missed step was scored “0”; the patients who scored “8” were considered using appropriate Rotahaler technique and were excluded from the FFTTR. The patients who failed to demonstrate the correct technique (*n* = 20) were provided with individualized instruction and training till no error was made by the patients [verbal instruction followed by demonstration through placebo (twice) and face-to-face training]. During the pharmacist's instruction, the patients were also allowed to ask questions for further clarification. The baseline assessments, education, and training were carried out by the first pharmacist on the same day of visit to the hospital. Following the face-to-face training, the patients were reinstructed about the technique through telephone on a weekly basis for 2 weeks and were reminded on correct Rotahaler technique focusing on incorrect steps. Patients were also allowed to ask questions in case of any confusion and their assurance of understanding of the telephonic instruction was considered as the confirmation of their understanding of the correct Rotahaler technique. This conversation lasted for up to 5 minutes. The patients were then invited for reassessments of the technique after 4 weeks of the training obtained in their first encounter at the Medication Counseling Centre. The reassessments of technique were performed by second pharmacist (not involved in the initial assessments) using the same checklist [18]. A pre-/postscore comparison was used to evaluate the effectiveness of FFTTR for improving Rotahaler technique [4, 18, 21]. Descriptive statistics including Wilcoxon Signed Rank test was applied for statistical analysis. A *p* value of <0.05 was considered as significant. Data were analyzed using IBM-SPSS 20.0 (IBM Corporation, Armonk, NY, USA).

## 3. Results

The mean age of the patients was 66.06 ± 10.6 years. Females were predominant (70%) in this study. The median (interquartile range [IQR]) duration of Rotahaler use was 4 (2–6) years. About two-thirds of patients were illiterate. Majority of patients (29, 96.7%) received instruction for the use of Rotahaler technique and most of them (19, 65.5%) received instructions from pharmacy personnel. Verbal instruction (14, 48.3%) and combination of verbal instruction plus physical demonstration (14, 48.3%) were the most common methods of instruction. Just above two-fifths (13, 44.8%) of the patients received repeated instructions on use of Rotahaler technique. The median (IQR) frequency of hospital visit was 6 (3–13) times for the last 12 months. More than three-quarters (32, 76.6%) of patients received at least one course of antimicrobial treatment for the last 12 months. Eleven patients (36.7%) visited emergency department at least once for the last 12 months due to exacerbation of symptoms and ten (33.3%) patients were hospitalized (Table 1). Ten patients (33.3%) performed the Rotahaler technique correctly on baseline assessment and they were not considered for analyzing the effect of FFTTR. The total patients who received FFTTR were twenty.

The FFTTR (*n* = 20) corrected the Rotahaler technique in 18 (90%) patients and the median (IQR) score increased from 6 (5-6) before intervention to 8 (8-8) after intervention. This difference was statistically significant at *p* < 0.001 (Table 2).

At baseline assessment most of the patients (16, 80%) failed to breathe out gently before inhalation and hold breath for 10 seconds after inhalation (18, 90%). These errors were significantly improved after FFTTR and just one patient (5%) failed to perform step 4 (breathe out gently but not towards the inhaler mouthpiece) correctly (Table 3). Similarly, at baseline there were also errors in critical step 1 (hold Rotahaler vertically) (4, 20%) and critical step 3 (split capsule into cap and body) (4, 20%) both of which improved by 15% after FFTTR.

## 4. Discussion

Correct inhaler technique is crucial for ensuring better treatment outcomes and for the control of disease [3] and is basically driven by the quality of initial instruction provided to the patients for appropriate use of inhaler by HCPs [22]. Our study revealed that two-thirds of the patients with COPD were using their Rotahaler device incorrectly. Most of them had failed to breathe out gently before inhalation and hold breath for about 10 seconds after inhalation. Similar findings have been reported by other studies [7, 18]. Failure in the aforementioned steps can cause low lung deposition of drug particularly in children and those with severe airflow limitation [23]. Error was also observed in critical step 1 (hold Rotahaler vertically) and critical step 3 (split capsule into cap and body) among one-fifth of the patients in our study. It is essential to hold the Rotahaler at an angle lesser than 45° from the vertical while inserting the capsule (step 1) to prevent the retention of drug in the opening in the back of the inhaler after splitting the capsule (step 3) [20]. Poor inhaler technique is one of the most common reasons for suboptimal management of COPD [3, 24, 25]. Moreover, it increases the risk of hospitalization, frequency of emergency visit, and courses of oral steroids and antimicrobials [3, 8]. Our study also showed that more than three-quarters of patients received at least one course of antimicrobial treatment, more than one-third of patients visited emergency department, and one-third of patients were hospitalized due to exacerbation of symptoms. These results might be the consequence of improper inhaler technique by the patients. The improper inhaler use can be attributed to issues related to device, patient, physician and healthcare professional, practicality, and cost [26].

The FFTTR achieved the correct use of the Rotahaler technique in 90% (18 out of 20) of the patients, at least one-fifth higher than results obtained by previous methods of instruction (3.3%–70%) [4, 6, 17–19]. Additionally, two of the critical steps (step 1 and step 3) also improved from 80% to 95% after FFTTR. These suggest that combination of face-to-face training and telephonic reminder can improve the inhaler technique in greater number of patients than achieved by other methods of instruction. Evidences from systematic reviews suggest that patients with COPD, particularly those with severe forms, are associated with considerable cognitive impairment [27, 28]. Therefore, telephonic reminder may play an indispensable role to recall the technique and this might be responsible for the achievement of promising results observed in our study. Furthermore, FFTTR may reduce the need of regular training and reassessments of technique by HCPs in this group of patients and is more effective in resource limited setting. Patients who have financial constraints for visiting hospital regularly would benefit particularly. Telephonic assessment and education of inhaler technique by pharmacist have shown significant improvement of inhaler technique [29] and patients were satisfied with home telecare [30–32]. Moreover, telemonitoring of patients with COPD lowers the rates of hospital admission, emergency department attendance, and interval of hospital readmission, together with cumulative length of stay [30, 33]. In addition, telemonitoring has also been known to be useful for treating acute exacerbation of COPD at home [34]. Therefore, telepharmacy offers several advantages such as easy access to healthcare services in remote and rural areas, financial benefits, patient satisfaction, effective patient counseling, and minimal scarcity of local pharmacist and pharmacy services [35].

Our pilot study evaluated the value of FFTTR for improving Rotahaler technique in patients with COPD in Nepal for the first time and results are convincing. Implementation of this service in hospital and community pharmacies can provide hope for improvement of inhaler technique in such groups. This service will be practically useful in resource limited setting like Nepal and for those patients who have financial limitation to visit hospital for a regular check-up. However, the effectiveness of telephonic reminder using a single pre-/postinterventional design with no control group and without considering Hawthorne effect [36] might not be ideal. Although our pilot study was able to provide preliminary findings, the generalizability of this study would need further elaboration warranting a larger controlled trial.

## 5. Conclusion

The patients with COPD receiving FFTTR as a specialized pharmacy service at Chitwan Medical College Teaching Hospital had marked improvement in their Rotahaler technique. This approach can be considered superior to the previous methods of instruction (physical demonstration; combination of verbal instruction and physical demonstration; combination of verbal counseling, physical demonstration, and video demonstration; and combination of verbal instruction, physical demonstration, and face-to-face training) in our setting. This pilot study should be scaled up in larger population to evaluate the impact of FFTTR for improving inhalers technique in similar group of patients.

## Figures and Tables

**Table 1:** Baseline characteristics of the patients assessed for the intervention (*n* = 30).

Variables	Category	*n* (%)
Age (years)^*∗*^		66.06 ± 10.62

Sex	Male	9 (30)
	Female	21 (70)

Education status	Illiterate	20 (66.7)
	Literate	10 (33.3)

Duration of Rotahaler use (years) ^*∗∗*^		4 (2–6)

Previous instructions on Rotahaler technique	Yes	29 (96.7)
	No	1 (3.3)

Source of instructions (*n* = 29)	Pharmacy personnel	19 (65.5)
	Clinicians/physicians	9 (31.0)
	Nurses	1 (3.4)

Method of instructions (*n* = 29)	Physical demonstration	1 (3.4)
	Verbal instruction	14 (48.3)
	Verbal instruction plus physical demonstration	14 (48.3)

Repeated instructions (*n* = 29)	Yes	13 (44.8)
	No	16 (55.2)

Number of hospital visits for the last 12 months^*∗∗*^		6 (3–13)

Antimicrobial courses for the last 12 months	None	7 (23.3)
	1	9 (30.0)
	2-3	10 (33.3)
	>3	4 (13.3)

Emergency department visits for the last 12 months	None	19 (63.3)
	1	9 (30.0)
	2-3	2 (6.7)

Hospitalization/hospital admissions for the last 12 months	None	20 (66.7)
	1	6 (20.0)
	2-3	3 (10)
	>3	1 (3.3)

^*∗*^Mean ± standard deviation. ^*∗∗*^Median [interquartile range (IQR)].

**Table 2:** Effect of FFTTR on correct use of Rotahaler technique (*n* = 20).

Assessment of Rotahaler technique	Median (IQR)	*p* value
Before intervention	6 (5-6)	<0.001^*∗*^
After FFTTR	8 (8-8)	

^*∗*^Wilcoxon Signed Rank test.

**Table 3:** Comparison of correct Rotahaler technique at baseline assessment and after FFTTR (*n* = 20).

Steps of Rotahaler technique	Baseline assessment	After FFTTR
	*n* (%)	*n* (%)
^*∗*^ *Step 1*. Hold Rotahaler vertically	16 (80)	19 (95)
*Step 2*. Put capsule into square hole	16 (80)	19 (95)
^*∗*^ *Step 3*. Split capsule into cap and body	16 (80)	19 (95)
*Step 4*. Breathe out gently but not towards the inhaler mouthpiece	4 (20)	19 (95)
*Step 5*. Put mouthpiece between lips and teeth	20 (100)	20 (100)
^*∗*^ *Step 6*. Breathe in the powder quickly and deeply	20 (100)	20 (100)
*Step 7*. Take Rotahaler device out of mouth before expiring	18 (90)	20 (100)
*Step 8*. Hold breath for about 10 seconds	2 (10)	20 (100)

^*∗*^ *Critical step*.
